# Ca^2+^ homeostasis: a potential target for cancer therapies

**DOI:** 10.52601/bpr.2024.230023

**Published:** 2024-10-31

**Authors:** Min Su, Shanliang Zheng, Hao Liu, Tie-Shan Tang, Ying Hu

**Affiliations:** 1 School of Life Science and Technology, Harbin Institute of Technology, Harbin 150001, China; 2 Key Laboratory of Science and Engineering for the Multi-modal Prevention and Control of Major Chronic Diseases, Ministry of Industry and Information Technology, Department of Medicine and Health, Zhengzhou Research Institute of Harbin Institute of Technology, Zhengzhou 450000, China; 3 State Key Laboratory of Membrane Biology, Institute of Zoology, Chinese Academy of Sciences, Beijing 100101, China; 4 Beijing Institute for Stem Cell and Regenerative Medicine, Beijing 100101, China; 5 University of Chinese Academy of Sciences, Chinese Academy of Sciences, Beijing 100101, China

**Keywords:** Calcium homeostasis, Cancer, Therapy

## Abstract

Calcium ions (Ca^2+^) play a crucial role as secondary messengers in both excitable and non-excitable cells. A complex system of proteins and molecules involved in calcium handling allows Ca^2+^ signals to be transduced. In cancer cells, mutations, aberrant expression, and dysregulation of these calcium handling toolkit proteins disrupt the normal Ca^2+^ flux between extracellular space, cytosol, endoplasmic reticulum and mitochondria, as well as the spatio-temporal patterns of Ca^2+^ signalling. This leads to the dysregulation of calcium-dependent effectors that control key signaling pathways involved in cancer cell proliferation, survival and invasion. Although there has been progressing in understanding the remodelling of calcium homeostasis in cancer cells and identifying key calcium transport molecules that promote malignant phenotypes, much work remains to be done to translate these fundamental findings into new tools for diagnosing and treating cancer by targeting Ca^2+^ homeostasis.

## INTRODUCTION

As the most versatile second messenger *in vivo*, calcium ions (Ca^2+^) are involved in a wide variety of cellular and physiological processes, including proliferation, cell death, migration, gene transcription, muscle contraction, *etc*. (Fiorio Pla and Gkika 2020). Notably, accumulating evidence over the past decade has shown that dysregulation of Ca^2+^ homeostasis is an important driving force in malignant transformation, tumour progression and angiogenesis (Marchi *et al.* 2020). Here, we focus on advances in the understanding of Ca^2+^ signalling behaviour and its role in tumour initiation, progression and treatment.

Under physiological conditions, Ca^2+^ concentrations in cells are maintained in a stable state with a gradient difference of Ca^2+^ by a series of Ca^2+^ channels and pumps. The cytosolic Ca^2+^ concentration needs to be maintained at a low level (∼100 nmol/L), approximately 10^4^-fold lower than the extracellular milieu (Marchi *et al.* 2020). In addition to the exchange with the extracellular Ca^2+^, there are also several Ca^2+^ storage sites within the cell, such as the endoplasmic reticulum (ER) (∼0.5 mmol/L) (Kim *et al.* 2021), Golgi (∼200 μmol/L) (Lissandron *et al.* 2010), lysosomes (∼500 μmol/L) (Zhong *et al.* 2017) and mitochondria (∼100–200 nmol/L) (Giorgi *et al.* 2018b). Under the control of a complex set of Ca^2+^ pumps, channels, and exchangers distributed at either plasma membrane or organelle membranes, changes in cytosolic Ca^2+^ or Ca^2+^ transport between organelles are perceived as specific signals to activate downstream responses that affect cell fate.

**Figure 1:**
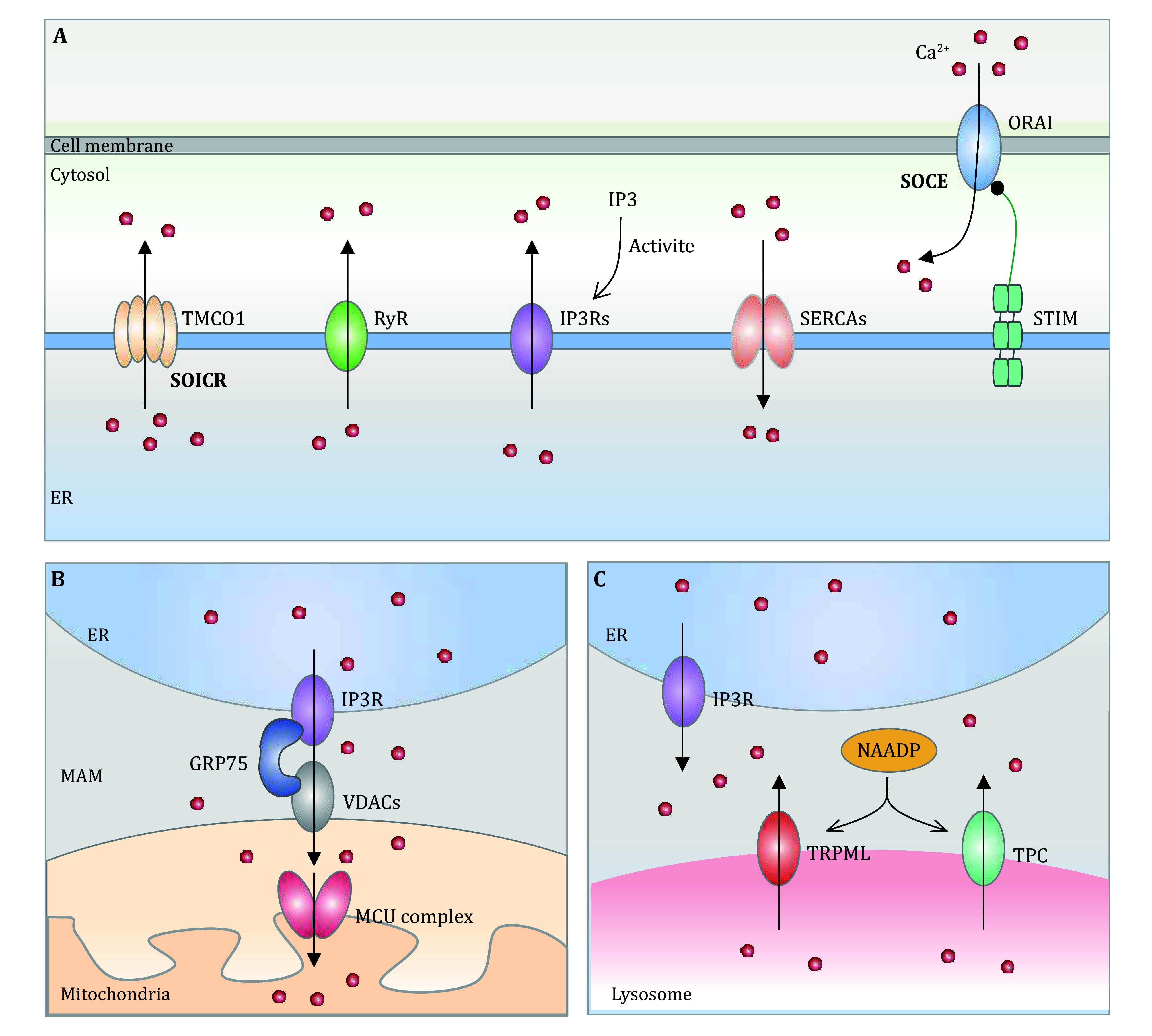
A graphical representation of calcium homeostasis attainment. **A** ER-Cytosol-Plasma membrane communications. ER-resident calcium pump proteins SERCAs are responsible for transporting Ca^2+^ from the cytosolic into the ER. IP3Rs, or RyRs mediate the release of calcium from the ER in response to the increased IP3 following hormone or growth factor stimulation, or increased cytosolic Ca^2+^ levels. When ER Ca^2+^ concentration is reduced, STIM can be activated to interact with and activate plasma membrane-localized Ca^2+^ channel ORAI1, leading to extracellular Ca^2+^ being influx and the subsequent SERCA-mediated ER Ca^2+^ refilling. Nevertheless, during ER Ca^2+^ overload, the TMCO1 can be stimulated to release Ca^2+^ into the cytosol by forming a selective Ca^2+^ channel through monomer-to-tetramer transformation, a process termed SOICR. **B** ER-Mitochondria communications. The ER and mitochondria are interconnected both structurally and functionally through a specialized microdomain known as the MAM. Within MAMs, several proteins such as Grp-75, VDAC1, and IP3R play a crucial role in regulating Ca^2+^ release from the ER and an efficient mitochondrial Ca^2+^ uptake. **C** ER–lysosome communications. Lysosomal Ca^2+^ depletion initiates the IP3R-mediated Ca^2+^ release from ER and subsequent lysosome Ca^2+^ refilling through the Ca^2+^ transporter or Ca^2+^/H^+^ exchanger (CAX). Furthermore, ER can restore Ca^2+^ from the lysosomes through the stimulation of two distinct mechanisms: the activation of TPC by NAADP, or the activation of TRPML1

Intracellular Ca^2+^ homeostasis is controlled by Ca^2+^ release from organelles or influx from extracellular spaces. ER-located Ca^2+^ channels, inositol 1,4,5-trisphosphate receptors (IP3Rs) or ryanodine receptors (RyRs), are responsible for ER Ca^2+^ release in response to elevated IP3 levels following hormone or growth factor stimulation or increased extracellular Ca^2+^ influx (Zheng *et al.* 2023). In response to ER Ca^2+^ depletion, the ER-resident protein stromal interaction molecule 1 (STIM1) is activated and subsequently interacts with and activates the plasma membrane-resident Ca^2+^ channel ORAI1, leading to extracellular Ca^2+^ influx, a process termed store-operated Ca^2+^ entry (SOCE) (Benson and Trebak 2023). On the other hand, sarcoplasmic endoplasmic reticulum-type Ca^2+^ ATPases (SERCAs), which are ER-resident Ca^2+^ pumps, are able to pump cytosolic Ca^2+^ back into the ER. During ER Ca^2+^ overload, the ER Ca^2+^ channel protein transmembrane and coiled-coil domains 1 (TMCO1) can be activated to release Ca^2+^ into the cytosol by forming a Ca^2+^ selective channel through monomer-to-tetramer transformation, a process termed store overload-induced Ca^2+^ release (SOICR) (Fig. 1A) (Sumitomo 2016).

In addition to the modulation of cytosolic Ca^2+^ to maintain intracellular Ca^2+^ homeostasis, Ca^2+^ transports between organelles, mainly ER-mitochondria or ER-lysosome, are also essential. For example, the mitochondria-associated ER membrane (MAM) is the interface between the ER and mitochondria and is involved in several biological processes, mainly by modulating ER-mitochondrial Ca^2+^ transport (de Ridder *et al.* 2023). It has been shown that ER-resident IP3R and mitochondria-localized voltage-dependent anion channels (VDACs) form the Ca^2+^ passage to transport Ca^2+^ from the ER to mitochondria, resulting in enhanced mitochondrial Ca^2+^ uptake across the mitochondrial outer membrane (Sander *et al.* 2021). The subsequent Ca^2+^ influx into the mitochondria was enforced by a Ca^2+^ gradient through the mitochondrial calcium uniporter (MCU) located across the inner mitochondrial membrane (Fig. 1B) (Murphy and Steenbergen 2021). In addition, it is worth noting that ER can replenish their Ca^2+^ stores from the lysosomes through the activation of two different pathways: the nicotinic acid adenine dinucleotide phosphate (NAADP)-evoked activation of two-pore channels (TPC) or the activation of mammalian mucolipin TRP channel subfamily (TRPML1) (Capel *et al.* 2015; Marchant *et al.* 2022; Rosato *et al.* 2021). There are numerous calcium-exchange pathways that play a crucial role in the regulation of calcium homeostasis that have not been enumerated in this review (Fig. 1C).

In short, normal cells maintain calcium homeostasis and modulate calcium signalling through a complex and interconnected system that operates in different cellular compartments. This system allows Ca^2+^ to act as a second messenger while preventing its potentially harmful effects. Conversely, cancer cells exhibit a number of abnormalities in this machinery, providing an opportunity for the development of innovative therapeutic agents, as discussed below (Zheng *et al.* 2023).

## Ca^2+^ HOMEOSTASIS DYSREGULATION AND CARCINOGENESIS IN CLINICAL SETTINGS

Cancer progression may be influenced by abnormal Ca^2+^ influx. Clinical evidence suggests that hypercalcaemia is often associated with the occurrence of malignant cancer. This condition has been shown to promote the growth and spread of gastric, oesophageal (Anderson *et al.* 2019), cervical (Lei *et al.* 2022), breast (Das *et al.* 2020) and colorectal cancers (Anderson *et al.* 2019) and has been considered a hallmark of end-stage disease. In colorectal cancer, the intestine has the ability to absorb significant amounts of calcium through specific ion channels, leading to hypercalcaemia in many patients. Many recent findings suggest that even a small increase in intracellular calcium signalling may contribute to the development and progression of colorectal cancer, while sustained calcium influx and overload can lead to tumour cell death (Villalobos *et al.* 2017). This dual function of calcium signalling adds complexity to the understanding of colorectal cancer and may offer new avenues for clinical intervention. In addition, in prostate cancer, non-small cell lung cancer and colon cancer, moderate levels of Ca^2+^ contribute to tumour progression, while excessive cytoplasmic Ca^2+^ levels also cause cell death by stimulating downstream Ca^2+^-dependent sensors (Cui *et al.* 2017; Silvestri *et al.* 2023).

In addition to cytosolic Ca^2+^ homeostasis, organellar Ca^2+^ homeostasis is also critical for tumour development and progression. For example, low levels of Ca^2+^ in mitochondria contribute to the growth of colon and non-small cell lung cancers by controlling energy production. Conversely, excessive Ca^2+^ accumulation in the mitochondria can also trigger cell death (Cui *et al.* 2017).

In conclusion, changes in Ca^2+^ fluxes affect a complex network of intrinsic (*e*.*g*. metabolism, redox homeostasis) and extrinsic (*e*.*g*. antigen presentation, danger signalling) processes in cancer cells, which in turn influence malignant transformation, tumour growth, and responses to therapy (Marchi *et al.* 2020; Monteith *et al.* 2017). Therefore, it is crucial to improve our understanding of the mechanisms by which perturbation of Ca^2+^ homeostasis leads to tumour progression.

## CALCIUM HOMEOSTASIS DISRUPTION AND CANCER HALLMARKS

**Figure 2:**
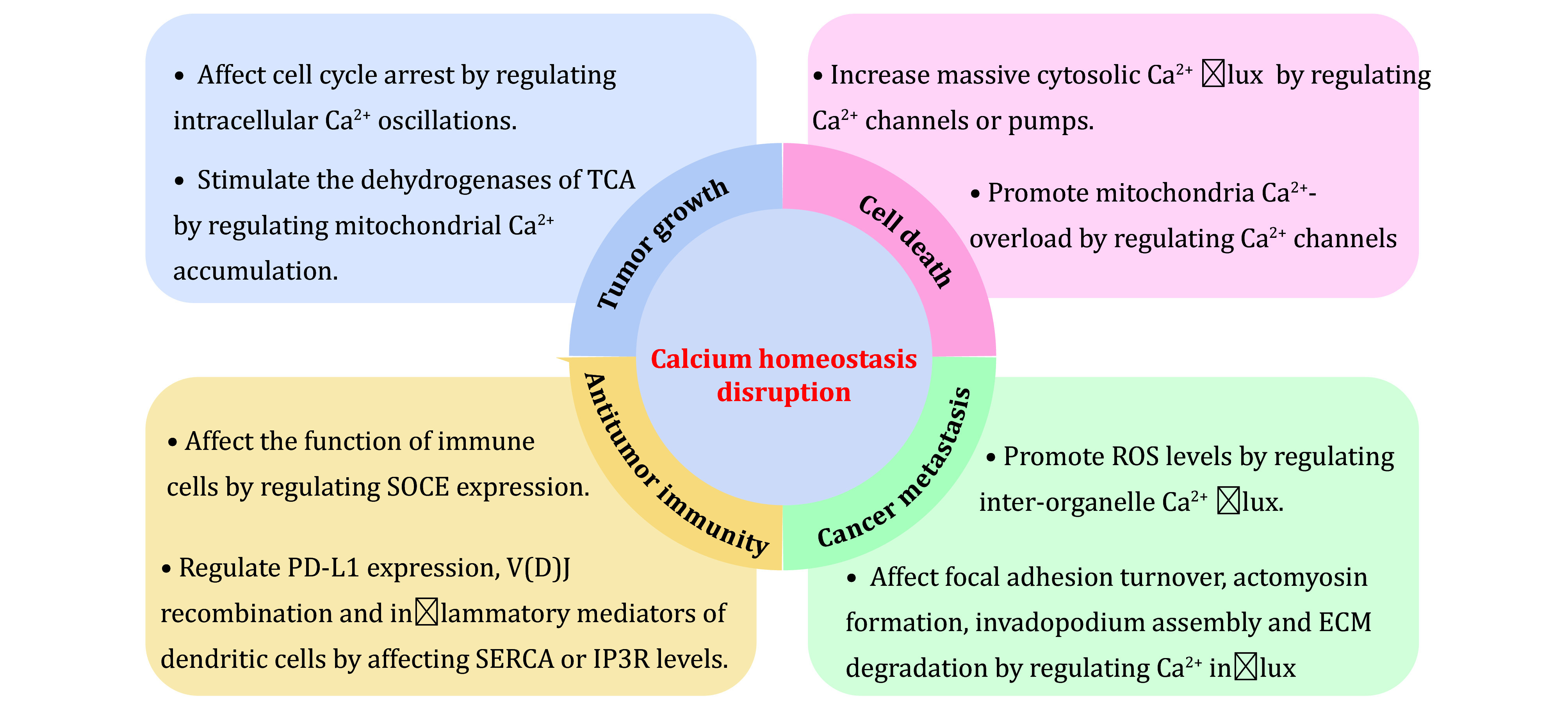
The relationship between calcium homeostasis disruption and cancer hallmarks. The examples demonstrate that disruption of calcium homeostasis is involved in mainly cancer characteristics, including tumour growth, cancer cell death, cancer metastasis, and antitumor immunity

The role of Ca^2+^ signalling in carcinogenesis and clinical status of cancer has been evidenced by the fact that altered Ca^2+^ homeostasis has been implicated in multiple cancer pathological processes. Further mechanistic study reveals that calcium homeostasis disruption can influence tumour growth, cancer cell death, metastasis and anti-immunity by modulating Ca^2+^ channels or pumps (Marchi *et al.* 2020) (Fig. 2).

### Calcium homeostasis disruption and tumour growth

Aberrant cell cycle progression is one of the fundamental mechanisms underlying tumourigenesis (Liu *et al.* 2022). Changes in cytosolic Ca^2+^ can regulate the cell cycle by altering Ca^2+^ transporters, consistent with the fact that Ca^2+^ signalling is involved in the regulation of cancer cell proliferation. Chaochu Cui *et al*. reported that Orai1, as a SOCE channel protein, can regulate intracellular Ca^2+^ oscillations to prevent cell cycle arrest and promote cancer cell proliferation (Cui *et al.* 2018). A novel SOCE channel inhibitor, RP4010, has been shown to mediate intracellular Ca^2+^ flux to arrest the cell cycle at the G_0_/G_1_ phase, ultimately inhibiting tumour growth in oesophageal cancer (Cui et *al.* 2018). In addition, genetic inhibition of STIM1, the other member of the SOCE channel, can significantly inhibit cell proliferation by preventing cell cycle arrest at the S and G/M phases in cervical cancer (Chen *et al.* 2011). Genetic inhibition of STIM1/Orai1 or RP4010 may act as an inhibitor of SOCE-mediated Ca^2+^ signalling in a context-dependent manner.

In addition, genetic silencing or pharmacological inhibition (caffeine, 2-APB and xestospongin C, XeC) of IP3R3, has been shown to inhibit cancer cell proliferation by preventing the intracellular Ca^2+^ elevation (Guerra *et al.* 2019; Szatkowski *et al.* 2010; Ueasilamongkol *et al.* 2020). Similarly, it has been shown that Ca^2+^-dependent lysosomal exocytosis plays an important role in cancer growth and chemoresistance (Buratta *et al.* 2020). Inhibiting this mechanism reduces the invasiveness and chemoresistance of sarcoma cells, whereas increasing lysosomal exocytosis improves invasiveness and drug resistance (Machado *et al.* 2015). TRPML1 is an important regulator of lysosomal exocytosis. Indeed, cholesterol recycling via TRPML1-mediated lysosomal exocytosis promotes the growth of oncogenic HRAS-driven cancer cells. TRPML1 deletion in cancer cells inhibits cholesterol transport from lysosome to the plasma membrane, which lowers their growth rate (Jung *et al.* 2019). These have been developed as a potential target to inhibit tumour growth.

Low-level Ca^2+^ transport from the ER to the mitochondria has been shown to be essential for the maintenance of tumour growth (Cárdenas *et al.* 2016; Garbincius and Elrod 2022). The resulting mitochondrial Ca^2+^ accumulation can stimulate the key dehydrogenases of the Ca^2+^-regulated tricarboxylic acid cycle, suggesting that it is critical for the biosynthetic and bioenergetic needs of cancer cells (Csordás *et al.* 2018; Zhao *et al.* 2019). Consistent with the above findings, abnormal expression of MAM or MCU channels has been shown to promote cell proliferation in lung and colorectal cancers by inducing mitochondrial Ca^2+^ uptake (Arif *et al.* 2014; Liu *et al.* 2020; Zeng *et al.* 2018).

### Calcium homeostasis disruption and cancer cell death

Low levels of Ca^2+^ are known to exert pro-survival functions by altering the cell cycle or promoting mitochondrial metabolism through Ca^2+^-dependent stimulation of TCA (tricarboxylic acid) cycle and OXPHOS (oxidative phosphorylation), but excessive cytosolic Ca^2+^ levels cause cell death (Loncke *et al.* 2021; Zheng *et al.* 2023). Indeed, increased Ca^2+^ influx from extracellular space through SOCE can promote Ca^2+^-induced apoptosis. Roberta Gualdani *et al*. reported that siRNA-mediated depletion of STIM1 can dramatically reduce cisplatin-induced ROS production by reducing Ca^2+^ entry and subsequently induce apoptosis in non-small cell lung carcinoma cells (Gualdani *et al.* 2019). The additional members of SOCE have been shown to elicit a similar role in prostate cancer cells. Downregulation of ORAI1 can protect cancer cells from apoptosis induced by several reagents, such as thapsigargin, oxaliplatin and tumour necrosis factor α (Flourakis *et al.* 2010).

Massive cytosolic Ca^2+^ flux, mainly from the ER, has been implicated in cancer cell death (Loncke *et al.* 2021). For example, Ki Cheong Park *et al*. showed that pharmacological inhibition of SERCA promotes Ca^2+^-dependent apoptosis during glucose deprivation in cancer stem-like cells (Park *et al.* 2018). Our data also show that TMCO1, as a SOICR channel protein, is overexpressed in colon cancer cells compared with normal controls (Zheng *et al.* 2022). Increased TMCO1 reduces Ca^2+^ levels in the ER store, which attenuates Ca^2+^ release from the ER to the cytosol, leading to the limited cytosolic Ca^2+^ dynamics upon drug treatment (TG and Stuarosporine) (Zheng *et al.* 2022). This mechanism may promote tumour growth and reduce staurosporine-induced apoptosis in colon cancer (Zheng *et al.* 2022). On the contrary, in bladder urothelial carcinoma, TMCO1 has been shown to have an opposite role, although whether its function is involved in ER Ca^2+^ regulation remains to be determined (Li *et al.* 2017). It is possible that it plays a different role in different cancer cells, depending on the context.

In addition, Ca^2+^ overload in the mitochondria also triggers cell death, mainly through Ca^2+^ transport from the ER to the mitochondria (Beaulant *et al.* 2022). For example, Xue Y showed that IP3R3 promotes apoptosis by enhancing Ca^2+^ transfer from the ER to the mitochondria, leading to the enhancement of cisplatin-induced apoptosis (Xue *et al.* 2021). Another MAM protein, VDAC1, has a similar function also by increasing mitochondrial Ca^2+^ influx (De Stefani *et al.* 2012). In conclusion, the promotion of massive cytosolic Ca^2+^ influx or mitochondrial Ca^2+^ overload has recently been proposed as a promising strategy to induce apoptosis in cancer cells.

### Calcium homeostasis disruption and cancer metastasis

Approximately 90% of cancer-related deaths are due to metastasis (Bakir *et al.* 2020). Metastasis consists of a number of processes, including the migration of cancer cells from their original growth sites to colonize distant organs (Gerstberger *et al.* 2023; Pastushenko and Blanpain 2019). SOCE, as a major influx route of intracellular Ca^2+^, can affect focal adhesion turnover during cancer cell migration, which was an early event in cancer metastasis. Indeed, Sang Kwon Lee *et al*. reported that decreased SOCE disrupted focal adhesion turnover and actomyosin formation by impairing Ca^2+^ influx in breast cancer cells (Lee *et al.* 2022). In addition, Jianwei Sun *et al*. showed that STIM1- and Orai1-mediated Ca^2+^ oscillations can increase invadopodium assembly and extracellular matrix (ECM) degradation, leading to the promotion of melanoma invasion, the later event in cancer metastasis (Sun *et al.* 2014). These findings provide a mechanism and shed new light on SOCE-mediated Ca^2+^ signalling in cancer cell metastasis.

In addition, cancer cell metastasis can be regulated by SERCA-mediated intracellular Ca^2+^ flux. As a result, phosphorylated cofilin failed to regulate F-actin polymerisation and lamellipodium formation, thereby impairing lamellipodium-based migration (Shi *et al.* 2018).

MAM-associated proteins are also involved in cancer cell metastasis by regulating inter-organelle Ca^2+^ flux. As a master gatekeeper gene that regulates Ca^2+^ transport from the ER to the mitochondria, VDAC1 contributes to cell migration by promoting ROS levels produced by the electron transport chain (Arif *et al.* 2014). In addition, Xiuchao Wang *et al*. have shown that another mitochondrial protein, MCU, promotes pancreatic ductal adenocarcinoma cell migration and invasion by activating the Keap1-Nrf2 antioxidant program (Wang *et al.* 2022).

### Calcium homeostasis disruption and antitumor immunity

SOCE-induced intracellular Ca^2+^ influx is essential for antitumour immunity, as abnormal SOCE proteins lead to the dysfunction of various immune cells, including T cells, B cells, natural killer (NK) cells, dendritic cells, mast cells, macrophages and neutrophils (Xie *et al.* 2016). Carl Weidinger *et al*. have shown that SOCE in CD8^+^ T cells is required to prevent the development of melanoma and colon cancer cells to control tumour growth (Weidinger *et al.* 2013). SOCE can regulate (cytotoxic T-lymphocyte) CTL degranulation, Fas ligand expression and TNF-α and IFN-γ production, which affect CTL cytotoxic function both *in vitro* and *in vivo* (Weidinger *et al.* 2013). In addition, antigen cross-presentation by dendritic cells (DCs) activates cytotoxic T-cell stimulation to enhance immunity against cancer (Nunes-Hasler *et al.* 2017). Deletion of SOCE impairs cross-presentation and DC migration by reducing Ca^2+^ signaling (Nunes-Hasler *et al.* 2017). Alternatively, lymphocytes mediate cytotoxicity by polarized release of cytotoxic granule contents towards their target cells (Maul-Pavicic *et al.* 2011). NK cells from SOCE-deficient patients exhibited defective SOCE and severely impaired exocytosis of cytotoxic granules, leading to impaired target cell lysis by reducing SOCE-mediated Ca^2+^ influx (Maul-Pavicic *et al.* 2011). These findings highlight an important role of SOCE in anti-tumour immunity and support the use of SOCE activity regulators in cancer therapy.

In addition, Ca^2+^ modulators are essential for anti-tumour immunity. Jun-Kyu Byun *et al*. showed that impaired SERCA activity can reduce cellular (Glutathione) GSH levels and subsequently upregulate PD-L1 expression under glutamine-limited conditions (Byun *et al.* 2020). This has been shown to suppress T cell-mediated anti-tumour activity (Byun *et al.* 2020). SERCA2 has also been reported to regulate V(D)J recombination. For example, Chun-Chin Chen *et al*. showed that mice with SERCA2-deficient B cells have decreased ER Ca^2+^ levels and increased cytosolic Ca^2+^ levels, resulting in a profound block in V(D)J recombination (Chen *et al.* 2021). Alternatively, IP3R-mediated Ca^2+^ mobilization can regulate NFAT activation, which suppresses cell survival and promotes the production of inflammatory mediators of dendritic cells (DC) (Marongiu *et al.* 2021). This suggests that Ca^2+^ mobilization may be a promising target for anti-inflammatory therapy or immunotherapies.

## THERAPEUTIC VALUES OF TARGETING Ca^2+^ HOMEOSTASIS

Chemotherapy, the basic cancer treatment, is beneficial for patients with early-stage tumours but is inevitably associated with the emergence of drug resistance during treatment (Bukowski *et al.* 2020). Therefore, elucidating the mechanism of action of chemotherapeutic drugs may provide a better basis for improving the sensitivity of chemotherapeutic drugs to improve the prognosis of patients. Although chemotherapy drugs have multiple mechanisms of action, many drugs have the function of regulating Ca^2+^ homeostasis, which is achieved by regulating Ca^2+^ modulators. For example, paclitaxel, cisplatin and doxorubicin, which are common chemotherapy drugs, increase the cytosolic Ca^2+^ levels or promote Ca^2+^ overload in mitochondria, thereby enhancing Ca^2+^-induced apoptosis (Varghese *et al.* 2019).

In addition to the function of chemotherapeutic drugs in regulating Ca^2+^ homeostasis, the combination of Ca^2+^-related regulators and chemotherapeutic drugs to kill tumours has also come to our attention. Given the important role of Ca^2+^ modulators in cancer development, a number of Ca^2+^ activators or inhibitors have been developed. For example, Celastrol, the curcumin analogue F36, JQ-FT and stemphol, which are SERCA inhibitors, are able to target SERCA and inhibit its activity, thereby inducing apoptosis (Fan *et al.* 2014; Ji *et al.* 2018; Roti *et al.* 2018; Xu *et al.* 2020). In addition, inhibitors that target calcium channels have also been shown to kill tumours by inhibiting cell metastasis or increasing apoptosis *in vitro* or *in vivo*, including the imidazole derivative SKF-96365 (Yang *et al.* 2009), a SOCE inhibitor, Synta66 (Waldherr *et al.* 2020), an ORAI1 inhibitor, the IP3R blocker 2-aminoethoxydiphenyl borate (2APB) (Waldherr *et al.* 2020). Moreover, the combination of some FDA-approved inhibitors with chemotherapy drugs has also shown promising effects in cancer treatments. For example, amlodipine, an FDA-approved Ca^2+^ channel blocker, can significantly enhance the therapeutic response of tumor cells to gemcitabine (Principe *et al.* 2022). Alternatively, Heejin Lee reported that Ca^2+^ channel inhibitors (manidipine, lacidipine, benidipine and lometzine) screened from the FDA-approved compound library show a clear inhibitory effect on the growth of ovarian (cancer stem cells) CSCs (Lee *et al.* 2020). Other chemotherapeutic agents have been shown to regulate Ca^2+^ signalling and exert anti-cancer effects, although the underlying molecular mechanisms require further investigation.

**Figure 3:**
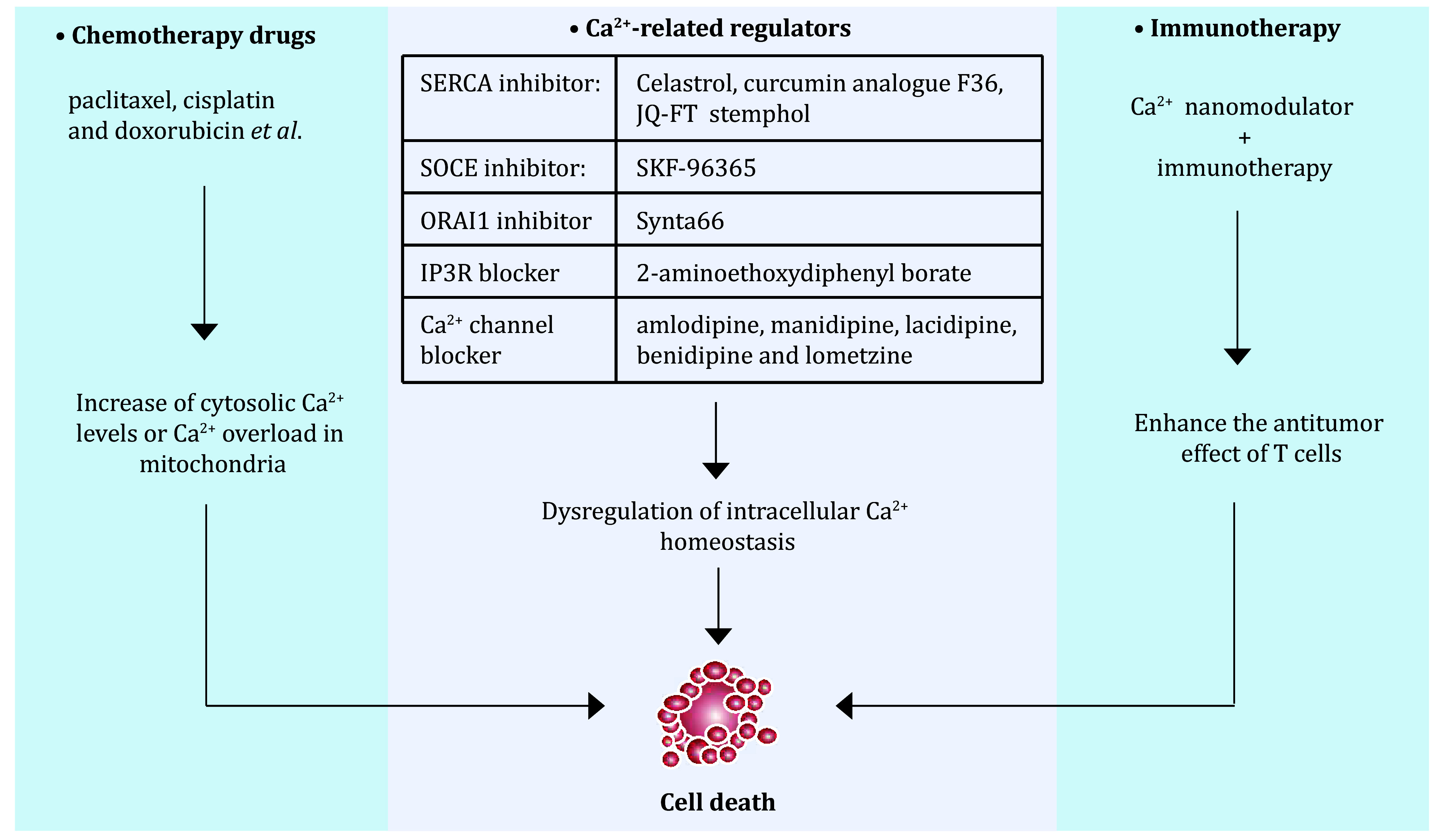
Targeting Ca^2+^ homeostasis as a potential therapeutic value. Relationship between targeted calcium homeostasis and tumor therapy, which contains Ca^2+^-related chemotherapy, combined-therapy and immunotherapy

Immunotherapy has become one of the most promising tumour treatment strategies in advanced cancer (Sharma *et al.* 2017). As mentioned above, activation of Ca^2+^ channels is necessary for the immune response, while dysregulation of Ca^2+^ homeostasis can lead to immune cell evasion in cancer cells (Shi *et al.* 2013). Therefore, based on the research on Ca^2+^ and tumour characteristics, it is reasonable to design a clinical strategy that combines the regulation of Ca^2+^ homeostasis with immunotherapy to achieve better therapeutic effects. It is worth noting that an ongoing project has used nanotechnology to deliver cytotoxic doses of Ca^2+^ into the tumour, followed by a combination with immunotherapy, which has so far shown promising anti-tumour effects (Zhao *et al.* 2021; Zheng *et al.* 2021) (Fig. 3).

### REMARKS

Ca^2+^ homeostasis is a necessary factor in maintaining normal growth and development of normal cells (Garbincius and Elrod 2022). Intracellular Ca^2+^ homeostasis requires a variety of Ca^2+^ channels and transporters to be maintained (Giorgi *et al.* 2018a). It is obvious that the imbalance of Ca^2+^ homeostasis caused by the abnormality of these proteins, is one of the major culprits that cause cells from a normal physiological state to a pathological state, resulting in the occurrence of various diseases, including cancer (Zheng *et al.* 2023). Regulating Ca^2+^ homeostasis was a double-edged sword for cancer cells. Modulating Ca^2+^ levels can promote tumour growth by interfering with cell cycle arrest. Conversely, massive cytosolic Ca^2+^ or mitochondrial Ca^2+^ overload promotes cell death (Marchi *et al.* 2020). It is therefore crucial to increase our knowledge of the mechanisms by which Ca^2+^ modulator functions lead to such opposite cellular outcomes.

Increased understanding of the role of Ca^2+^ homeostasis has greatly advanced the field of cancer, and Ca^2+^-based cancer therapeutics have attracted increasing attention. Although common chemotherapeutic drugs targeting Ca^2+^ have not yet been developed, more and more studies have shown that a variety of chemotherapeutic drugs can regulate Ca^2+^ homeostasis and thereby affect the fate of cancer cells (Varghese *et al.* 2019). In addition, several inhibitors of Ca^2+^ modulators screened from the FDA's library of compounds can be combined with chemotherapeutic drugs to kill cancer cells. These suggest that disrupting Ca^2+^ homeostasis to promote cancer cell death has become a remarkable effect. What's more, Ca^2+^-based therapy combined with immunotherapy has become a hot topic, while the underlying molecular mechanism needs to be further explored.

## Conflict of interest

Min Su, Shanliang Zheng, Hao Liu, Tie-Shan Tang and Ying Hu declare that they have no conflict of interest.
